# Beyond Noise: Using Temporal ICA to Extract Meaningful Information from High-Frequency fMRI Signal Fluctuations during Rest

**DOI:** 10.3389/fnhum.2013.00168

**Published:** 2013-05-01

**Authors:** Roland N. Boubela, Klaudius Kalcher, Wolfgang Huf, Claudia Kronnerwetter, Peter Filzmoser, Ewald Moser

**Affiliations:** ^1^Center for Medical Physics and Biomedical Engineering, Medical University of ViennaVienna, Austria; ^2^MR Centre of Excellence, Medical University of ViennaVienna, Austria; ^3^Department of Statistics and Probability Theory, Vienna University of TechnologyVienna, Austria; ^4^Department of Psychiatry and Psychotherapy, Medical University of ViennaVienna, Austria; ^5^Department of Radiodiagnostics and Nuclear Medicine, Medical University of ViennaVienna, Austria

**Keywords:** resting-state fMRI, temporal ICA, heart rate variability, resting-state networks

## Abstract

Analysis of resting-state networks using fMRI usually ignores high-frequency fluctuations in the BOLD signal – be it because of low TR prohibiting the analysis of fluctuations with frequencies higher than 0.25 Hz (for a typical TR of 2 s), or because of the application of a bandpass filter (commonly restricting the signal to frequencies lower than 0.1 Hz). While the standard model of convolving neuronal activity with a hemodynamic response function suggests that the signal of interest in fMRI is characterized by slow fluctuation, it is in fact unclear whether the high-frequency dynamics of the signal consists of noise only. In this study, 10 subjects were scanned at 3 T during 6 min of rest using a multiband EPI sequence with a TR of 354 ms to critically sample fluctuations of up to 1.4 Hz. Preprocessed data were high-pass filtered to include only frequencies above 0.25 Hz, and voxelwise whole-brain temporal ICA (tICA) was used to identify consistent high-frequency signals. The resulting components include physiological background signal sources, most notably pulsation and heart-beat components, that can be specifically identified and localized with the method presented here. Perhaps more surprisingly, common resting-state networks like the default-mode network also emerge as separate tICA components. This means that high-frequency oscillations sampled with a rather T1-weighted contrast still contain specific information on these resting-state networks to consistently identify them, not consistent with the commonly held view that these networks operate on low-frequency fluctuations alone. Consequently, the use of bandpass filters in resting-state data analysis should be reconsidered, since this step eliminates potentially relevant information. Instead, more specific methods for the elimination of physiological background signals, for example by regression of physiological noise components, might prove to be viable alternatives.

## 1 Introduction

The investigation of BOLD fluctuations in the resting brain using fMRI has been a rapidly expanding field of research since the first identification of consistent patterns in these data (Biswal et al., 1995), and ICA in particular has gained great popularity in fMRI as a powerful tool for exploring these data (Biswal and Ulmer, 1999; Calhoun et al., 2001). The appeal of Independent Component Analysis (ICA) in the context of resting-state fMRI (rs-fMRI) lies to a great extent in the fact that, in contrast to task-fMRI, little *a priori* knowledge about the temporal dynamics of the fluctuations is available and ICA can be used to identify consistent patterns in an exploratory manner (Beckmann, 2012). Thus, using ICA on rs-fMRI data, several consistent resting-state networks have been identified in a multitude of different individual studies (Damoiseaux et al., 2006; Robinson et al., 2009; Allen et al., 2011; Yeo et al., 2011) as well as in collections of data pooled from multiple sites (Biswal et al., 2010; Kalcher et al., 2012).

A common feature to most rs-fMRI ICA studies thus far is the use of relatively long TRs (usually 2–3 s) in order to increase BOLD weighting (Kim and Ogawa, 2012), and scan durations of mostly between 5 and 10 min (Biswal et al., 2010), limiting the fluctuations that can be studied to those at frequencies between 0.001 and 0.25 Hz. Within this frequency range, the highest amplitudes of oscillations in resting-state networks in these studies have been observed in the lower part (<0.1 Hz), which lead to the general characterization of resting-state brain networks as networks of low-frequency fluctuations, typically between 0.01 and 0.1 Hz (Margulies et al., 2010; Yeo et al., 2011; Kalcher et al., 2012).

In recent years, simultaneous image readout (SIR) and multibanded (MB) EPI pulse sequences allowing simultaneous acquisition of multiple brain slices during a single EPI echo train have opened new opportunities for accelerating fMRI scans without sacrificing spatial resolution (Feinberg et al., 2010; Feinberg and Yacoub, 2012). The increased temporal resolution can be put to use in different ways. First, the higher sampling rate allows to perform new kinds of analysis methods, leading to a new view on low-frequency fluctuations, as exemplified by the identification of temporal functional modes (TFM) by Smith et al. (2012). On the other hand, the increase in temporal resolution without the need to limit image acquisition to a few slices can be harnessed to investigate higher-frequency fluctuations at whole-brain level. Of course, this will change the specific contrast from mainly BOLD-based to flow/perfusion-based (Kim and Ogawa, 2012).

It should be noted at this point that the focus on low-frequency BOLD fluctuations is not only due to technical limitations, but also motivated by the temporal delays involved in the hemodynamic response to neuronal activity. Indeed, the peak of the hemodynamic response to a particular stimulus – and thus of the BOLD signal – occurs 3–10 s after the underlying neuronal response (Aguirre et al., 1998; Cunnington et al., 2002). Thus, the BOLD signal can be seen as temporally smoothed in comparison with the neuronal activity, motivating the neglect of signal fluctuations in higher frequencies. Nonetheless, the possibility to obtain this high-frequency signals opens the question to investigate what patterns can be found in these frequency domains.

Due to limited *a priori* knowledge on networks of high-frequency rs-fMRI BOLD oscillations, an exploratory approach seems most viable (Tukey, 1977) to get an unbiased estimation of the global structure of these oscillations. While different exploratory analysis techniques for fMRI data exist, e.g., principal components analysis (Baumgartner et al., 2000), canonical correlation analysis (Friman et al., 2001), fuzzy clustering (Baumgartner et al., 1998; Moser et al., 1999), as well as spatial or temporal ICA (Calhoun et al., 2001), our analysis specifically needs a method that can deal with overlapping spatial distributions of different signal sources. Temporal ICA (tICA) can achieve this in identifying temporally independent signal sources with potentially overlapping spatial distributions, and in this offers good interpretability, since its result is a solution to the blind source separation problem. In particular, the potential to better distinguish spatially overlapping signal sources might prove useful for the identification of cardiac and other physiological signal sources, a feature that spatial ICA cannot accomplish as shown by Beall and Lowe (2010).

Temporal ICA has rarely been used thus far in fMRI analyses, mostly due to two reasons. The first lies in originally unsurmountable computational difficulties in computing the necessary linear algebra operations, in particular computing the covariance matrix of dimension (number of voxels × number of voxels) (Calhoun et al., 2001), but new algorithms as well as the increased computational power available have greatly alleviated this limitation. The second reason is the limited number of time points (the data points for tICA) available in most fMRI scans, limited by common TRs of 2–3 s and scan durations under 10 min to about 300 time points. In contrast to spatial ICA, where the corresponding variable is the number of voxels instead of the number of time points, this limited amount of data points leads to computational issues regarding the stability of the ICA algorithm when applying it as temporal ICA. Multiplexed EPI sequences, with greatly reduced TRs, lead to larger amounts of data points without increasing scan duration, and thus allow for a reasonable application of tICA on the resulting datasets.

Beyond the increase in stability of tICA estimation, the high sampling rate also allows to see fluctuations of higher frequencies than before in whole-brain fMRI datasets. It is however unclear as of now what exactly is gained by critically sampling higher frequencies (at low TR). In this study, we set out to investigate the information gained in these high frequencies, and in particular the frequency domain above the highest frequency usually inspected in resting-state fMRI studies, about 0.25 Hz. *A priori*, two thoughts on these high-frequency fluctuations come to mind: first, they could be expected to contain pulsation-related artifacts, and second, due to the slow hemodynamic response usually expected for neuronal activity, one might be tempted not to expect to identify neuronal signals among the high-frequency BOLD oscillations. Indeed, early investigations by Cordes et al. (2001) on the relative contributions of different frequency ranges – Cordes et al. acquired signal from 4 slices with a TR of 400 ms – found that functional connectivity was almost exclusively dependent on the signal fluctuations below 0.1 Hz for neuronal signal sources, and only the correlation coefficients from signal in major arteries or veins as well as in the CSF were dependent upon higher frequencies.

However, there is some evidence in more recent studies that this latter expectation might not hold true. For once, studies on spectral characteristics of resting-state networks by Niazy et al. (2011) and Van Oort et al. (2012) have revealed that the spectral range of commonly identified resting-state networks is wider than the hypothesized 0.01–0.1 Hz and extend to at least 0.17 and 0.25 Hz, respectively. Moreover, there are studies on specific high-frequency behavior of BOLD oscillations, e.g., the co-occurrence of spikes in different regions of a particular network (Tagliazucchi et al., 2011, 2012) or variation in amplitude variance asymmetry (Davis et al., 2013), that can also be attributed to resting-state network activity, indicating consistent patterns of BOLD and/or perfusion variability beyond low-frequency fluctuations.

In this study, we investigated high-frequency signal fluctuations during rest by temporally filtering fMRI data with a low TR to frequencies above 0.25 Hz and analyzing the resulting timecourses using temporal ICA. In view of the hypotheses mentioned above, we examined the extent to which tICA is able to specifically separate physiological background signals, in particular heart-beat related signal fluctuations, from other signal sources in the brain, as this is seen as one of the “killer applications” of ICA in rs-fMRI (Beckmann, 2012). Moreover, we wanted to explore whether resting-state network related signals are still present in those high-frequency domains and could effectively be identified.

## 2 Materials and Methods

### 2.1 Subjects

Ten subjects (5 males/5 females, mean age 23.4, SD 3.1 years) were recruited at Medical University of Vienna. Exclusion criteria were prior psychiatric or neurologic illnesses, as well as the usual exclusion criteria for MR studies. All subjects gave written informed consent prior to the scan and the study was approved by the local institutional review board.

### 2.2 Measurements

Subjects underwent a 6 min resting-state scan on a Siemens TIM Trio 3 T scanner using a 32-channel head coil with a multiplexed EPI sequence by Feinberg et al. (2010), acquiring in total 1024 volumes (flip angle = 30°, TE/TR = 32/354 ms, 2.4 mm × 1.9 mm × 3.5 mm, bandwidth = 1748 Hz/pixel, 20 axial slices, 2 mm slice gap, multiband acceleration factor 4, 6/8 partial Fourier). Subjects were instructed to keep their eyes closed, refrain from movement during the scan and avoid to fall asleep without concentrating on anything in particular. After the resting-state scan, a high-resolution anatomical image was acquired using MPRAGE with 1 mm × 1 mm × 1.1 mm resolution with 160 sagittal slices (TE/TR = 4.21/2300 ms, flip angle 9°, inversion time 900 ms).

### 2.3 Preprocessing

All data were preprocessed with a combination of AFNI (Cox, 1996) and FSL (Smith et al., 2004), using an analysis framework in R (Boubela et al., 2012; R Development Core Team, 2013) on Ubuntu Linux (Version 11.10 “Oneiric Ocelot”). Anatomical images were skullstripped and normalized to MNI152 standard space. Functional images were corrected for intensity inhomogeneity using a bias field estimation by FSL FAST, skullstripped and realigned to the 500th volume. Subsequently, functional images were aligned to the anatomical images in MNI152 standard space and resampled to 2 mm × 2 mm × 2 mm isotropic resolution, blurred with an isotropic Gaussian 6 mm FWHM kernel, and motion parameters (3 translations and 3 rotations) were regressed out using a generalized linear model (GLM).

### 2.4 Independent component analysis

After the preprocessing steps mentioned above, all further analyses were performed in R (Version Under Development (unstable) 2012-11-27 r61172 “Unsuffered Consequences”; this version was used to allow the allocation of objects with more than 2^31^ – 1 elements, necessary for the processing of time concatenated group ICA). At single-subject level, the first 24 volumes of all subjects were discarded to account for transient effects, and all voxel time-series were scaled to mean 0 and standard deviation 1. To isolate high-frequency oscillations, a discrete Fourier transform was applied to each voxel’s time course, all magnitudes in Fourier space corresponding to frequencies below 0.25 Hz (the highest frequency that can be sampled at a typical TR of 2 s) were set to 0, and the signal was then transformed back in the original space using the inverse discrete Fourier transform. Thus, the signal that was analyzed contained only fluctuations above 0.25 Hz. Single-subject data were analyzed individually as well as concatenated for group analysis, forming a 10,000 (i.e., 10 subjects × 1000 time points) × 239901 (number of voxels within the brain mask) matrix. Prewhitening and dimensionality reduction was performed by principal component analysis (PCA) using the R package irlba (Baglama and Reichel, 2005, 2012), which implements implicitly restarted Lanczos bidiagonalization singular value decomposition (SVD), and the 76 principal components with the largest eigenvalues were computed and used for the ICA analysis. All matrix multiplications on the data matrix necessary to compute the SVD and the principal components were performed using the library phiGEMM (Spiga and Girotto, 2012), which distributed computation on two NVidia Tesla C2070 graphics processing units. Finally, fastICA (Hyvärinen, 1999) was used to compute 75 temporally independent components for the time concatenated group dataset.

### 2.5 Group components

**Figure A1:**
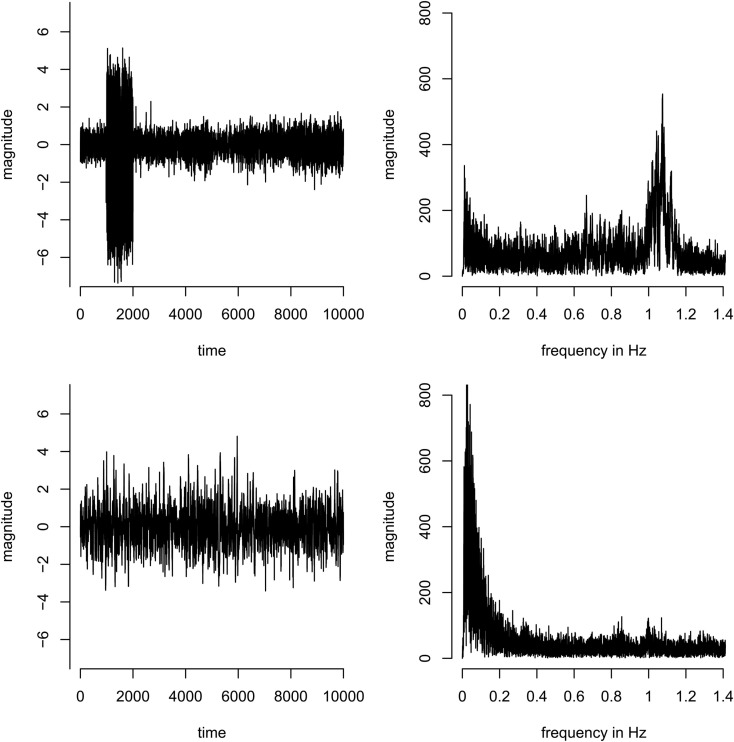
**Concatenated time-series (left) and corresponding spectra (right) of two example components**. Top: a component dominated by a single subject – most of the variance of the time course originates from subject 2 (time points 1001–2000 in the concatenated time-series). Bottom: a component that is equally present in all subjects, i.e., the variance in the concatenated time course is more homogeneous across subjects.

In the group analysis, components were discarded if they were driven by individual subjects only (as opposed to being present in all subjects; this can easily be identified in the component timecourses, see Figure A1 in Appendix). As a formal criterion, components were discarded if the ratio of the sum of the squares of the time course of one subject divided by the sum of the squares of the time courses of all other subjects was larger than 1, i.e., if one subject contributed more variance to the component than all other subjects combined.

### 2.6 Characterization of resulting components

Spatial maps of all resulting components were projected back from the principal component space into the original space. Temporal ICA time courses were Fourier transformed to compute power spectra, and the fraction of the power in each of the frequency ranges 0.25–0.5, 0.5–0.75, 0.75–1.0, 1.0–1.25, and 1.25–1.4 Hz was computed.

### 2.7 Low-frequency reference networks

To get a sense of how resting-state networks obtained in the high-frequency range relate to low-frequency resting-state networks, the data preprocessed as above but without applying the high-pass filter were analyzed with temporal ICA directly and the resulting networks were used as reference for the high-frequency networks.

## 3 Results

Of the 75 tICA components, 25 were found to be consistent across subjects using the definition above, i.e., no single subject contributed more to the component than all other subjects combined. Among these consistent group-level components, four distinct types of components can broadly be distinguished: pulsation or physiological components (8 components), components resembling known resting-state networks as described by previous low-frequency sICA studies (2), technical artifacts (2), and other signal sources (13).

**Figure 1:**
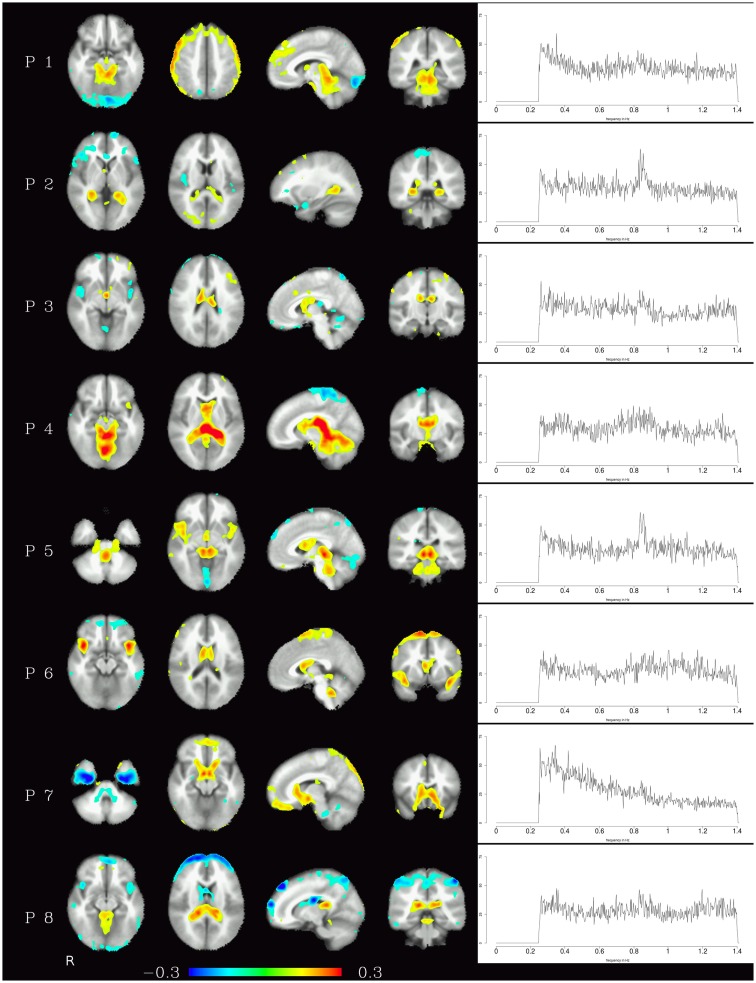
**Temporal ICA components attributed to pulsation in ventricles and large blood vessels**. Left: maps thresholded at 0.1 (weights in the mixing matrix). Right: frequency spectra corresponding to the ICA components represented on the left.

Generally speaking, pulsation components were the most consistent across subjects using the measure described above. They were located primarily in the ventricles and in the vicinity of large blood vessels (see Figure 1 left) and exhibited more amplitude in higher frequencies (mainly above 0.6 Hz, see Figure 1 right). Specifically, the ventricular components had peak power between 0.6 and 0.8 Hz, while other pulsation components including mainly the insula had a broader frequency range between 0.6 and 1.4 Hz. Overall, though, it can be said that pulsation artifacts showed a flat, modulated power spectrum.

**Figure 2:**
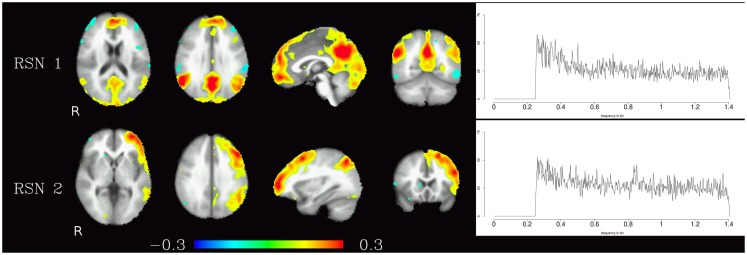
**Temporal ICA components representing high-frequency fluctuations in brain regions commonly associated with resting-state networks**. Figure layout as in Figure 1.

The resting-state components identified in the high-frequency range were the default-mode network and the fronto-parietal network, the corresponding maps are shown in Figure 2. In contrast to the pulsation artifacts, resting-state network timecourses tended to have higher amplitude in the lower frequencies (0.25–0.6 Hz). The most distinctive characteristic of the spectra of the resting-state networks as opposed to the pulsation and artifact components is their skewness – amplitude is highest for lower frequencies and decreases continually as the frequency increases, and converges to a minimum at about 0.6 Hz.

**Figure 3:**
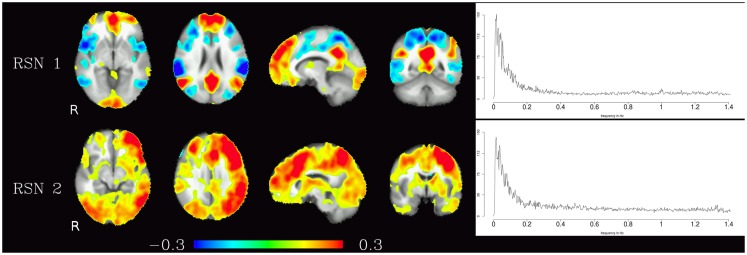
**Temporal ICA components from non-bandpassed data corresponding to the high-frequency resting-state networks in Figure 2**. Figure layout, color scale, and threshold are identical to the ones in Figure 2.

**Figure 4:**
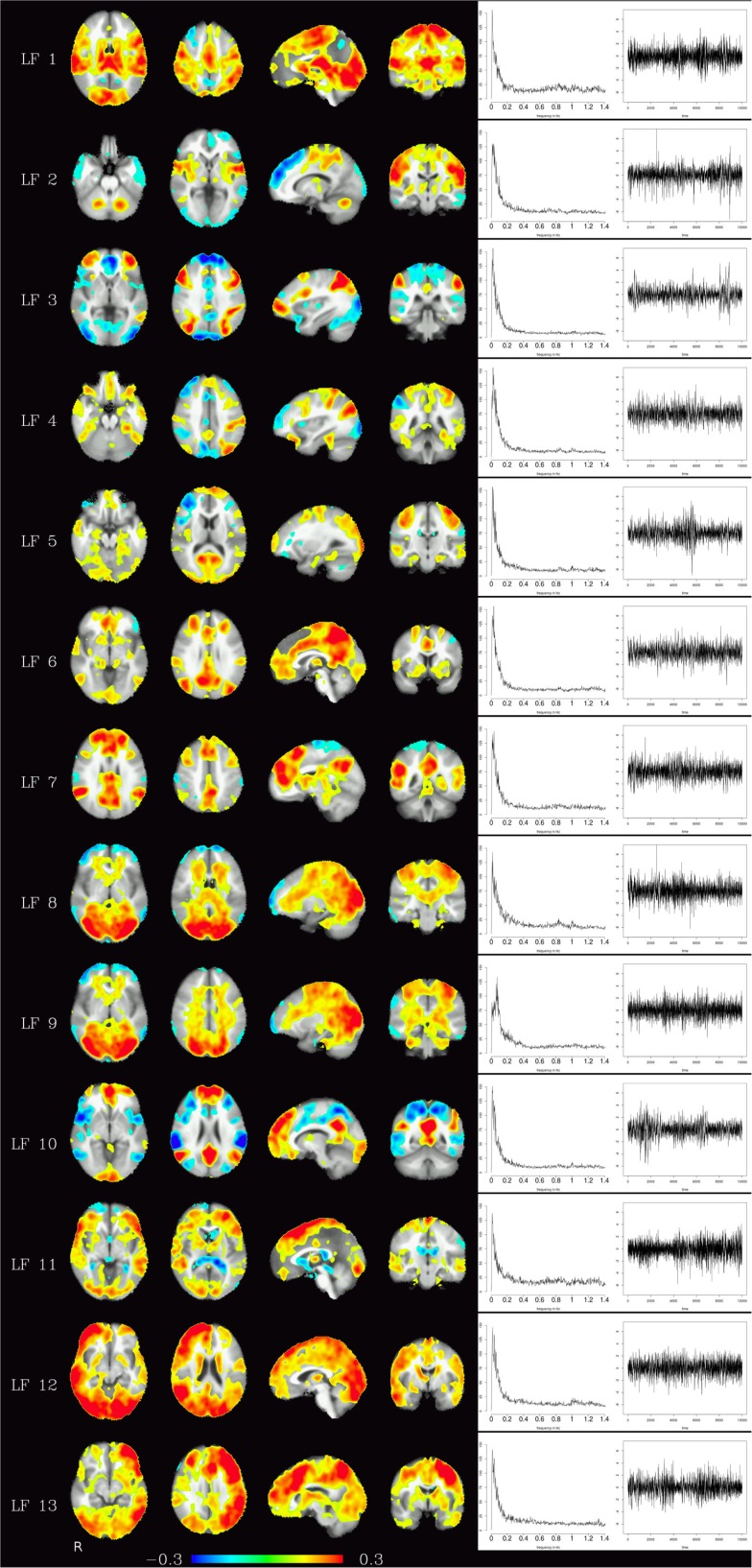
**Depending on the number of components chosen, various temporally independent low-frequency components (≤0.25 Hz) are separated by the algorithm (LF 1–LF 13, left row)**. Note that time courses and corresponding frequency spectra (right side) are not contaminated by any high-frequency components (e.g., respiration, heart-beat, etc.), increasing functional contrast-to-noise ratio. The interpretation whether a component is (predominantly) of vascular or brain tissue origin, however, is not obvious from the spectra alone.

Corresponding resting-state networks could also be identified in the analysis of the non-bandpassed data (Figure 3). It can be seen that most of the power of the resting-state networks originates in the low-frequency range (below about 0.2 Hz), but the qualitatively very similar maps in the high-frequency data suggest that these same networks can also be identified by their distinctive high-frequency fluctuations, which indeed amount to about 50% of the total spectral power of these networks. Table 1 summarizes the fraction of power of the fluctuations of these networks that fall in the frequency bands 0.01–0.1, 0.1–0.25, and 0.25–1.4 Hz. (For further reference, Figure 4 shows the complete set of components identified by tICA on the unfiltered data.)

**Table 1:** **Fractional amplitude of fluctuations in various frequency bands (0.01–0.25, 0.01–0.10, 0.10–0.25, 0.25–1.4 Hz) for RSN 1 and RSN 2 depicted in Figure 3**.

	0.01–0.25 Hz (%)	0.01–0.10 Hz (%)	0.10–0.25 Hz (%)	0.25–1.4 Hz (%)
RSN 1	46.77	31.24	15.52	52.49
RSN 2	49.91	33.87	16.04	49.3

**Figure 5:**
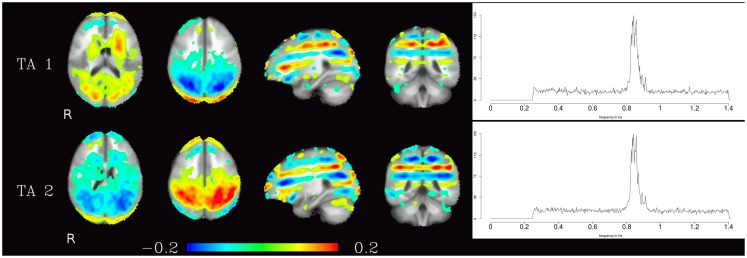
**Temporal ICA components attributed to technical artifacts**. Figure layout as in Figure 1, spatial maps are thresholded at 0.05. Note that even though they cover almost the whole brain, and thus have at least some overlap with all other components, tICA is able to separate them from the other components due to the technical artifacts distinctive temporal characteristics (visible in their power spectra).

The third group of components were technical artifacts defined by two unique characteristics. The first emerges from the spatial maps of these components, which shows alternating bands of high and low loadings aligned in planes parallel to the acquisition slices (see Figure 5 left). The second characteristic is the narrow peak of the frequency spectrum at about 0.8 Hz (see Figure 5 right).

**Figure 6:**
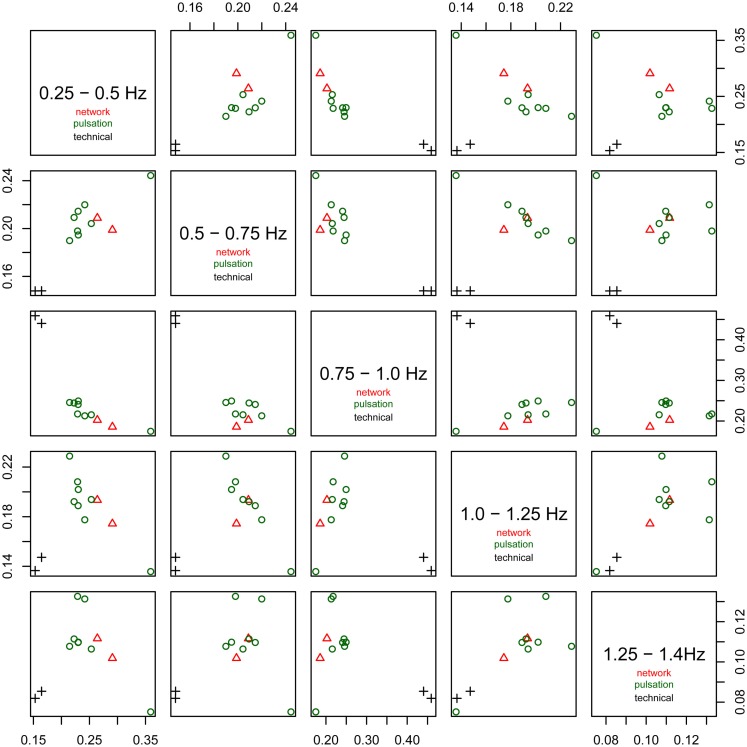
**Fractional amplitude of fluctuations in frequency bands 0.25–0.5, 0.5–0.75, 0.75–1.0, 1.0–1.25, and 1.25–1.4 Hz plotted against each other for all consistent tICA components**. Note that technical artifacts can easily be separated from all other components in the frequency bands 0.25–0.5, 0.5–0.75, and 0.75–1.0 Hz. Components attributed to classical resting-state networks appear as mixed with pulsation components, though they tend to have higher power in the lowest frequency range, between 0.25 and 0.5 Hz, than most of the pulsation components.

The relative power of each frequency range (0.25–0.5, 0.5–0.75, 0.75–1.0 Hz, 1.0–1.25 Hz, and 1.25–1.4 Hz) of the spectra is shown in Figure 6. The technical artifacts are easiest to distinguish due to their power being almost entirely in the range between 0.75 and 1.0 Hz, with much higher relative power in this range than all other components, and very low power in all other frequency bands. Resting-state networks can also be distinguished by their having highest relative power in the lowest of the frequency bands (0.25–0.5 Hz), while the pulsation components and other artifacts have lower power in this frequency range, but tend to have higher power in all other ranges. Overall, the distribution of relative spectral power is more similar between resting-state networks and pulsation components than between any one of these groups and technical artifacts.

**Figure 7:**
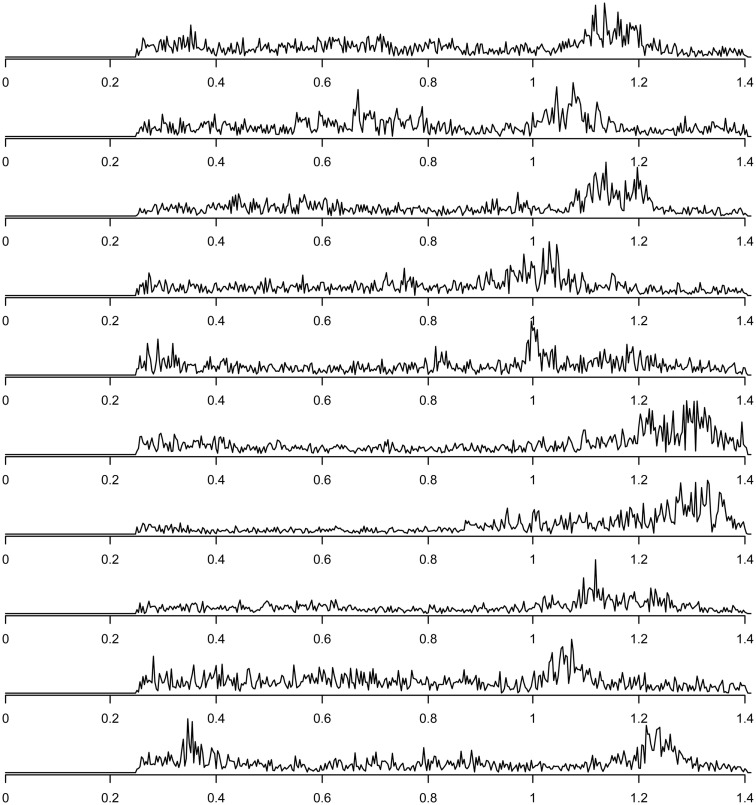
**Frequency spectra of components attributed to heart-beat (with peaks in the frequency range around 1–1.3 Hz), one component for each of the 10 subjects**.

Finally, components related to heart-beat could be found in the components discarded due to their inconsistency across subjects (this inconsistency presumably is due to heart rate differences between subjects). For each subject, the spectrum of the group component driven mainly by that subject that can be interpreted as heart-beat related signal is shown in Figure 7. The power spectra of these heart-beat components can be distinguished by their peak at frequencies around 1–1.3 Hz (the exact frequency of the peak varies, depending on the heart rate variability (HRV) of the individual subject). Thus, HRV would be a physiological parameter to be extracted from our data.

## 4 Discussion

In this work, we have shown that consistent large-scale high-frequency signal oscillations in the brain exist and can be attributed to specific signal sources using temporal ICA. Potentially of most practical interest among these are the physiological or pulsation-related components and the resting-state networks, but other signal sources can be distinguished as well. We have concentrated on fluctuations of frequencies higher than 0.25 Hz to study consistent effects that cannot be identified in typical fMRI experiments with a TR of about 2–3 s, since they are beyond the Nyquist frequency of the measurements performed in these experiments. It should be noted that, even though they cannot be isolated when using TRs of 2–3 s, in the resulting data these high-frequency effects are nonetheless present in the form of aliased lower-frequency fluctuations, i.e., so-called physiological noise. The specific identification of pulsations and artifacts can be useful in disentangling them from neuronal signal sources, in order to isolate the latter more specifically, but also to study physiological effects by themselves.

Two innovations from different fields have been employed in this study in order to identify the high-frequency components of fMRI signal. First, the measurement of whole-brain time-series at the low TR required for a sufficiently high sampling rate has only become possible with the introduction of multiband EPI sequences (Feinberg et al., 2010; Moeller et al., 2010; Feinberg and Yacoub, 2012), allowing the simultaneous acquisition of multiple slices and leading to a reduction of the TR to 354 ms with the parameters used in this study. Second, new computational methods were needed to perform the analysis at hand. This included improvements in handling the large datasets generated by this sequence, with both high spatial and high temporal resolution, as well as fast iterative computation of SVD – and by consequence of PCA and ICA – on these datasets, both necessary to divide the signal acquired into temporally independent sources (Boubela et al., 2012).

Perhaps the most surprising finding of this study was the identification of resting-state networks, and most notably the default-mode network, in the high-frequency data alone. While the traditional view of resting-state networks as low-frequency fluctuations below 0.1 Hz has been challenged, previous findings have related mostly to oscillations below 0.16 (Niazy et al., 2011) and 0.25 Hz (Van Oort et al., 2012). The present study adds to this the notion that even in frequencies beyond the frequency range critically sampled by usual fMRI acquisition sequences, oscillations attributable to resting-state networks can be recognized. Indeed, the amount of information contained in the high-frequency oscillations of these two networks is sufficient to produce a spatial delineation consistent with previously published spatial maps, even despite the small sample size. Consequently, further investigations into the fluctuation characteristics of resting-state networks embracing the recent developments in fast fMRI acquisition techniques appear to be worthwhile. Indeed, whether ’sources of resting-state BOLD responses are similar to those of stimulus-induced responses’ is still an open question and part of ongoing research (Kim and Ogawa, 2012), and it is not yet clear if and to what extent the theory of hemodynamic coupling can be drawn upon to substantiate the widespread dismissal of high-frequency oscillations in resting-state fMRI.

The identification of resting-state networks in high-frequency data of course does not imply that they are primarily high-frequency phenomena, but rather that the frequency range of resting-state fluctuations is broader than previously assumed. Still, it must be noted that only two of the typically described resting-state networks were found in the high-pass-filtered data of this study. Both the default-mode network and the fronto-parietal network are characterized by high low-to-high-power ratio and high dynamic range (defined as the difference between the peak power of the spectrum minus the minimum of the power at higher frequencies compared to this peak) (Robinson et al., 2009; Kalcher et al., 2012), which seems paradoxical for networks that can be identified by their high-frequency oscillations. On the other hand, these two metrics are also associated with the robustness of the networks, i.e., networks with high power ratio and high dynamic range are identified more robustly across studies, and this robustness of the networks might be the reasons why only these two are identified here. High-frequency oscillations in other resting-state networks might exist, but in this case, their power must then be too low to be detected with the SNR level attained in this study.

The identification and separation of physiological signal sources made possible by the combination of a high sampling rate and temporal ICA of the resulting time courses can be seen as another way of using the high-frequency data and has multiple applications. First, the ability to disentangle physiological signal components from signals of neuronal origin could be used for the correction of the typical BOLD signal and thus for increasing the specificity not only of resting-state, but also of task-fMRI analyses (based on the assumption that physiological signals are the same during tasks and during rest). Correction of fMRI time-series for non-neuronal effects could then be performed using either the time course itself or a separately measured dataset (e.g., a resting-state dataset measured before or after a task-fMRI paradigm) (Kalcher et al., 2013). As another possible future application, measuring and separating physiological signals directly from fMRI data, as opposed to using separately acquired physiological respiratory and cardiac signals, would have the advantage that these signals could immediately be located in the brain using the spatial maps of the corresponding components, and would not require additional equipment for the acquisition of physiological signals. Indeed, the possibility of directly estimating cardiac and respiratory signal from the fMRI data has already been explored, e.g., by Beall and Lowe (2007) and Chuang and Chen (2001), and the methods presented here could be used to improve on these techniques. One potential advantage of avoiding the need for additional equipment is an increase in reliability of the complete system due to less individual parts that can possibly fail which might be critical for particular applications like real-time fMRI (Weiskopf, 2012). Furthermore, reducing the number of components separately introduced into the measuring systems means reducing the possible amount of operator bias – thus effectively increasing reproducibility of fMRI study results and comparability across studies in the face of possible future meta-analyses (Huf et al., 2011). Finally, direct measurement in the subject’s brain could circumvent time-delay issues due to measurement of multiple physiological variables on different parts of the body, e.g., the acquisition of pulse-oximetry data on the finger. Of course, these suggestions would require further studies to demonstrate their suitability for routine application.

Previous approaches taken to eliminate physiological signal sources include bandpass-filtering to frequencies below 0.1 Hz, but the adequateness of this method has been questioned – one of the main reasons for this being that many physiological confounds (like heart-beat) occur beyond the Nyquist frequency of typical measurement sequences and are thus aliased into the lower frequency ranges. On one hand, the higher sampling rate as used in this study avoids aliasing of high-frequency signals into lower frequencies, thus making the bandpass approach potentially better able to separate low-frequency from higher-frequency signals than it has been the case for long-TR measurements. On the other hand, this study highlights that a considerable amount of information on resting-state network activity pattern is lost when only looking into low-frequency fluctuations. This corroborates existing findings by Tagliazucchi et al. (2011, 2012) that as much as 50% of correlation patterns are lost when eliminating the information in the BOLD spikes they investigated. Furthermore, there is evidence that blood-flow related BOLD signal sources originating in the vessels of the brain are important confounding factors that should be taken into account specifically (Strik et al., 2002). Thus, the use of a bandpass filter to frequencies below 0.1 Hz is only advisable if one is explicitly interested in low-frequency dynamics alone, as opposed to studies investigating resting-state networks more generally.

While the range of applications mentioned above see the physiological components as signal of no interest to be eliminated from the data, it is equally possible to treat them as the main target for analysis. The identification of disruptions in the normal pattern of physiological fluctuations in the brain can be useful for clinical applications, for example in the localization of lesions (Yating et al., 2013), an application where the high temporal resolution can be critical for the detection of signal delays. Indeed, pulsations in the arteries of the brain have already been studied as main focus of research by Strik et al. (2002), and HRV would be a valuable parameter when studying patients with cardiovascular diseases.

Scientific implications of the results shown here might be that high-frequency signal oscillations should not be ignored, they can and should be measured with current acquisition techniques and should not be eliminated from analyses by coarse-grained correction methods such as bandpassing the entire fMRI time-series. Future investigations might focus on the development of more specific correction for physiological effects, if one attempts to eliminate those from the dataset, for example by using their tICA component time courses as regressors.

The findings presented here further challenge the traditional view of resting-state networks as low-frequency oscillations alone and support the idea of them exhibiting more complex behavior. Additional work on the temporal dynamics of resting-state network activity patterns might help to understand the structure of the brain processes underlying the associated BOLD and perfusion related fluctuations. In this study, only two resting-state networks could be consistently identified across subjects by their high-frequency components. This could be interpreted in terms of differences in spectral characteristics between resting-state networks, but could also be due to the scan duration used here (6 min) being insufficient to detect other networks. Future studies might uncover similar high-frequency components in other resting-state networks using longer scan duration or higher sampling rate with lower TRs and higher sensitivity, e.g., at 7 T.

## Conflict of Interest Statement

The authors declare that the research was conducted in the absence of any commercial or financial relationships that could be construed as a potential conflict of interest.
